# Automated phantom analysis for gamma cameras and SPECT: A methodology for use in a clinical setting

**DOI:** 10.1002/acm2.13057

**Published:** 2020-11-03

**Authors:** Tutku E. Tazegul, Andrew M. Polemi, Angela Snyder, Carl Snyder, Patricia G. Collins

**Affiliations:** ^1^ Atirix Medical Systems, Inc Minneapolis MN USA; ^2^ Department of Radiology and Medical Imaging UVA Health Charlottesville VA USA

**Keywords:** automated phantom analysis, gamma camera, nuclear medicine, quality control, SPECT

## Abstract

**Purpose:**

We introduce an automated, quantitative image analysis package for gamma camera and single photon emission computed tomography quality control. Our focus was to produce consistent methods that are feasible in clinical settings and use standard phantoms.

**Methods:**

Four gamma cameras were used to acquire planar images of four‐quadrant bar phantoms and projection views of an American College of Radiology (ACR) phantom as part of a standard gamma camera quality control program. Images were sent to QC‐Track® (Atirix Medical Systems, Inc., Minneapolis, MN, USA), which automatically placed predetermined regions of interest (ROIs) and performed analysis. For the bar phantom, a standard deviation (SD)‐based modulation transfer function was calculated for a circular ROI in each quadrant. The bar widths at various MTF values were reported using linear interpolation as applicable. For the ACR phantom, the contrast‐to‐noise ratio (CNR) for each sphere, a modulation for each rods section, and a percent deviation for uniformity ROIs was calculated. Spheres corresponding to a CNR of 3, and the rod size at various modulations were also reported using linear interpolation. Visual analysis was performed by three medical physicists to evaluate interobserver variability and correlation to quantitative values.

**Results:**

Analysis of the bar phantom showed predictable differences with changes in matrix size and bar width and showed consistency over similar acquisitions over the course of the study. Analysis of the ACR Phantom showed increasing CNR and modulation with increasing sphere and rod diameter, as expected. For both phantoms, quantitative values from linear interpolation correlated well with visual analysis.

**Conclusion:**

Our automated method for quantitative image analysis is consistent and shows increased precision and sensitivity when compared to standard visual methods. Thresholds correspond well with visual analysis and previous guidelines for observer visibility (e.g., Rose criterion), making our framework suitable for routine use in a nuclear medicine department.

## 1 INTRODUCTION

Consistent quality control (QC) is key to maintaining image quality in a nuclear medicine department. Routine assessment of a gamma camera’s energy peaking, planar intrinsic and extrinsic uniformity, intrinsic and extrinsic spatial resolution, and spatial linearity have been recommended by accrediting bodies such as the American College of Radiology (ACR)^1^ and the Intersocietal Accreditation Commission (IAC).^2^ For single photon emission computed tomography (SPECT) systems, additional tests using volumetric phantoms with different inserts are recommended to assess tomographic spatial resolution, cold sphere contrast detectability, and uniformity.

Energy peaking and planar uniformity testing for gamma cameras is often performed using manufacturer‐supplied software. For the remaining tests, there are a variety of phantoms and methodologies. For planar spatial resolution and linearity testing, many phantoms, such as slit, orthogonal hole, parallel line equal spacing, and bar, are commercially available. It has been previously noted that four‐quadrant bar phantoms are commonly used clinically due to their convenience^3^; they can be used in combination with a sheet or a point source to evaluate both intrinsic and extrinsic spatial resolution, as well as linearity. For evaluation of tomographic acquisitions (i.e., SPECT), the ACR‐approved Jaszczak phantom is used at many sites and is required for ACR accreditation.^4^ As such, both the four‐quadrant bar phantom and the ACR phantom are important tools for a complete clinical nuclear medicine QC program.

In the clinic, QC phantom images are often evaluated visually, subjecting the tests to inter‐ and intraobserver variability and bias.^5^ In some cases, knowledge of pass/fail thresholds and pressure to keep imaging units active for patient imaging may influence the evaluator to pass a test that may be borderline by visual assessment. Additionally, qualitative analysis of phantom images is insensitive to small degradations in image quality, and evaluation is limited to discrete the levels of assessment dictated by the physical design of the phantom. As a whole, visual analysis lacks precision and reproducibility, but has proven valuable for uniformity and artifact evaluations.^6^, ^7^

Conversely, automated, quantitative analysis ensures objectivity and consistency while increasing sensitivity. Historically, the AAPM has described standardized methods for performing QC tests and methods to quantify their results. AAPM Report 9 of the Nuclear Medicine Task Group described methods and standard definitions for quantifying integral and differential uniformity, statistical uniformity index, dead time, sensitivity, relative sensitivity (per collimator), spatial linearity, and the full‐width at half maximum (FWHM) of a line source.^6^ AAPM Report 22 further described a set of quantitative tests for gamma cameras with rotating heads.^8^ These include the tests of system alignment, collimator hole angulation, tomographic uniformity and contrast, and attenuation correction. AAPM Report 52 was designed to be a comprehensive performance testing program for SPECT.^9^ It provided testing instructions, quantification methods, and acceptable values for physicist tests of SPECT systems including rotational field uniformity and sensitivity, tomographic uniformity, spatial and contrast resolution, and attenuation correction. More recently, AAPM Report 177 included valuable updates and described methods suitable for clinical physics acceptance testing and annual evaluation of gamma cameras and SPECT units.^10^ It also included the descriptions of routine clinical quality control tests. This is the most current AAPM Report on SPECT/gamma camera QC.

Independently, others have created software and described their own quantitative metrics for gamma camera QC. Hasegawa et. al. presented software that analyzes a custom orthogonal hole phantom and volumetric flood source to evaluate spatial resolution, linearity, and uniformity.^11^ Hander et. al. described a method to measure gamma camera spatial resolution by using the mean and standard deviation of ROIs placed on a four‐quadrant bar phantom.^12^, ^13^ Madsen showed that annular sampling of a tomographic uniformity image provides a better statistical separation of images with and without ring artifacts compared to nonsampled methods.^14^

More recently, De Nijs et. al. presented a MATLAB‐based software to calculate NEMA NU‐1 2007 based quality control metrics.^15^ Nelson et. al. showed that noise texture analysis could be used to better assess planar uniformity floods compared to pixel‐based methods.^7^ Hirtl et. al. designed and built an ImageJ plugin which automatically places ROIs, performs SPECT contrast measurements for the Jaszczak phantom spheres and rods, and tests uniformity by means of a Hough transform or student’s t test.^16^ Nichols explored various image texture metrics to link qualitative statements of phantom sphere and rod visibility to quantifiable parameters, including count quantile metrics, gray‐level co‐occurrence matrix metrics, image contrast metrics, and count histogram metrics.^17^ DiFillipo tested software that evaluates contrast of planar ACR rods images, and further explored how creating receiver operating characteristic (ROC) curves for rods sections in the tomographic reconstructions of the ACR SPECT and PET phantoms can better classify rod visibility.^18^

While these methods are certainly viable and have demonstrated great improvements over a visual analysis, they are better suited for academic or research centers with ample resources, time, and the knowledge to implement these methods for QC. Most of these methods are not feasible for routine clinical evaluation and have not seen wide‐spread clinical implementation. Many of the methods presented previously either do not supply software to perform the appropriate calculations, utilize software not commonly found in most clinics (ImageJ, MATLAB, etc) require significant user input during analysis, demand some level of programming knowledge, or greatly increase the time required to perform QC. As a result, most clinical physicists and technologists choose not to adopt these methods and instead resort to visual analysis due to its efficiency.

The goal with this work was to create an automated QC workflow that is as efficient as visual analysis, and has the added benefits of objectivity, consistency, and sensitivity provided by quantitative analysis. Here we propose a method that is packaged to be easy to implement clinically not only for physicist use but also for technologist use, so the robustness of automated analysis can be applied across the QC program.

## 2 MATERIALS AND METHODS

### 2.A QC‐Track

QC‐Track® (Atirix Medical Systems, Inc., Minneapolis, MN, USA) is commercially available software for diagnostic imaging quality control that allows representative images of phantoms to be used to build standard phantom analysis templates. Quality control data for each imaging unit can then be saved within QC‐Track by sending images to the software via an image router or secondary destination. QC‐Track assigns images to the appropriate device and phantom template using information in the DICOM header and requires minimal user input through a web‐based user interface to save and report quality control data. All automated image analysis was performed in QC‐Track with the help of the clinical nuclear medicine technologists that typically perform QC in the department.

QC‐Track also allows data to be exported as.csv files for import into a researcher’s preferred analysis software. In this study, quality control data were exported from QC‐Track and analyzed with in‐house python scripts.

#### 2.A.1 Phantom and worksheet templates

Representative images of each phantom were used to build standard phantom templates. These templates were designed to read DICOM header information to correctly assign images, use image processing techniques to detect the phantom within each image, automatically place ROIs, and perform the appropriate calculations. To limit computational complexity, phantom templates were built using ROIs with a fixed position relative to the center of the phantom, and thus require a consistent phantom orientation during acquisition. Worksheet templates were built to automatically populate QC data from phantom images. We focused our initial efforts on designing templates for the bar and ACR phantoms as most gamma camera and SPECT manufacturers provide on‐board software to analyze flood uniformity and center of rotation test results. The phantom and worksheet templates for the ACR phantom were built such that the user can select which transaxial slice they would like to analyze for each section of the phantom.

##### Bar phantom

**Fig. 1:**
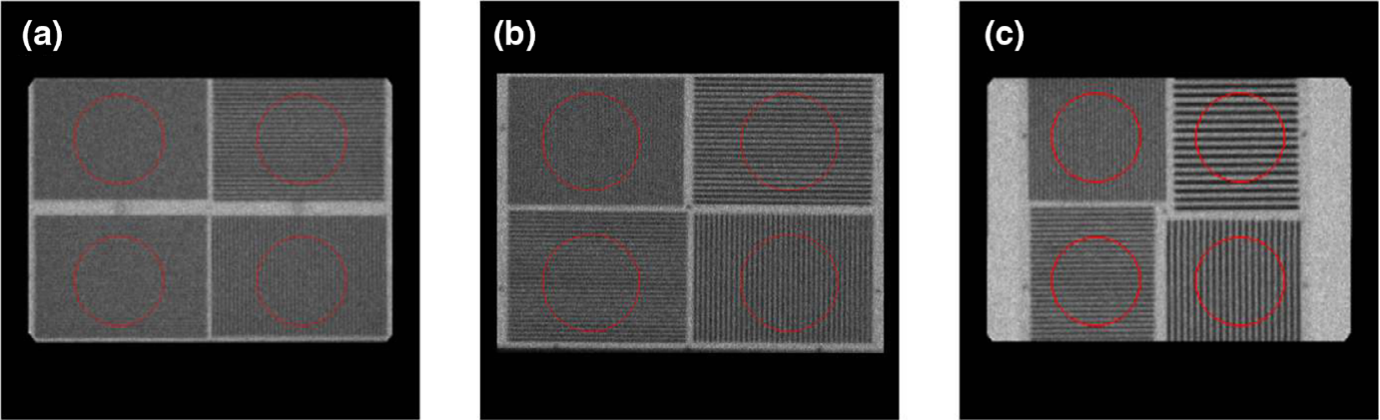
Bar phantom region of interest (ROI) placement for (a) bar phantom 1, (b) bar phantom 2, and (c) bar phantom 3. ROIs are shown in red.

For the bar phantom, three phantom templates were designed: one for rectangular phantoms with bar sizes 3.5, 3.0, 2.5, and 2.0 mm [“bar phantom 1” – Fig. 1(a)], one for rectangular phantoms with bar sizes 4.0, 3.5, 3.2, and 2.5 mm [“bar phantom 2” – Fig. 1(b)], and the third for square phantoms with bar sizes 6.4, 4.8, 4.0, and 3.2 mm [“bar phantom 3” – Fig. 1(c)]. All three templates were built to accommodate any square image matrix size (256 × 256, 512 × 512, etc.) by using information available in the DICOM header. All three templates apply a morphological dilation, followed by an erosion and Gaussian blur to eliminate small objects in the image and reduce noise. A threshold algorithm is used to detect the phantom and find the position of the phantom center (P_x_, P_y_). For bar phantoms 1 and 2, circular ROIs with 132 mm diameters are placed within each quadrant at locations (P_x_ ± 134 mm, P_y_ ± 104 mm). For bar phantom 3, circular ROIs with 132 mm diameters are placed within each quadrant at locations (P_x_ ± 106 mm, P_y_ ± 106 mm) due to the difference in the phantom shape.

##### ACR phantom

**Fig. 2:**
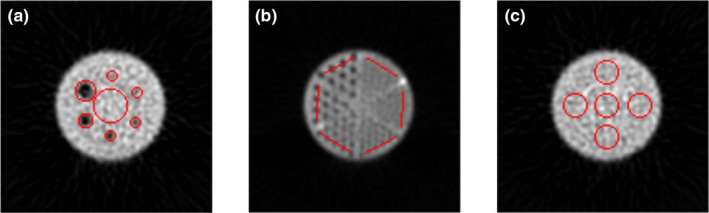
American College of Radiology single photon emission computed tomography phantom region of interest (ROI) placement for (a) spheres, (b) rods, and (c) uniformity sections. ROIs are shown in red.

For the ACR phantom, templates were designed for the rods, spheres, and uniformity sections. Like the bar phantom, the templates apply a morphological dilation, followed by an erosion to eliminate small objects in the image. A threshold algorithm is used to detect the phantom, as well as the position of the phantom center (P_x_, P_y_) for each axial slice. For the spheres section, a circular background ROI with a 69.2 mm diameter is placed at the phantom center (P_x_, P_y_). Circular ROIs are placed over the spheres [Fig. 2(a)]. For the rods section of the phantom, a single 72‐mm‐line ROI is placed over the outer set of rods for each section [Fig. 2(b)]. For both the spheres and rods sections, the geometric specifications of the phantom design were used to define the ROI placements within the template. For the uniformity test, five circular ROIs with 50 mm diameters are placed within the uniformity section of the phantom, mimicking uniformity evaluations performed in CT and PET [Fig. 2(c)].

### 2.B Computations

#### 2.B.1 Four‐quadrant bar phantom

Functions were built within the bar phantom templates to calculate a standard deviation (SD)‐based modulation transfer function (MTF) within each circular ROI as described previously.^13^ Additionally, a linear interpolation across the MTF values at the bar frequencies corresponding to the largest three bar widths was built to report the bar widths at MTF values of 0.15 and 0.1. From our initial tests, bars corresponding to an MTF of 0.15 correlated well with visual analysis, while an MTF value of 0.1 has been reported by Cherry et. al as an approximate value for visual resolvability.^19^ For bar phantom 3, a linear interpolation across the MTF values at the bar frequencies corresponding to the largest four bar widths was built to report the bar width at MTF values of 0.5 and 0.25 due to the larger bars of this phantom.

#### 2.B.2 ACR SPECT phantom

The ACR SPECT phantom contains sections for cold sphere contrast detectability, cold rod spatial resolution, and uniformity. The sections for cold sphere contrast detectability and cold rod spatial resolution include cold sphere inserts and cold rod inserts, respectively.

##### Spheres

For the spheres section, functions were built within the ACR phantom template to calculate a contrast to noise ratio (CNR) between each sphere and the central background ROI. The CNR equation was adapted from the contrast measurement for the spheres section described in both AAPM Report 52 and AAPM Report 177.^9^, ^10^ Additionally, a linear interpolation across the CNR values for the three smallest spheres was built within the template to report the lesion size corresponding to a CNR of 3, based on the lower limit of the Rose Criterion.^19^

##### Rods

Since the line ROIs for the rods section are placed along the outer set of rods, the count profile of the line ROI can be approximated as sinusoidal, and a pixel‐based modulation can be calculated across each ROI. For each axial slice containing the rods section of the phantom, the modulation in each rod sector was calculated. An aggregate modulation was also calculated by averaging the results across ten slices within the rods section. A linear interpolation across the modulations of the five largest rod sectors was built to report the hypothetical rod size that would correspond to modulation values of 0.1, 0.15, 0.2, 0.25, 0.3, 0.4, and 0.5. The rod sizes at these modulation values were then used for correlation to a visual analysis and to set a quantitative threshold on the modulation.

##### Uniformity

The template and calculations for the uniformity section were modeled from uniformity tests employed in CT. Manufacturer‐supplied uniformity phantoms, as well as the ACR CT phantom,^20^ are evaluated by comparing the mean pixel intensity of four peripheral ROIs to a central ROI. Typically, a percent deviation is reported for each peripheral ROI. That same test was replicated here.

### 2.C Clinical implementation and routine use

Clinical implementation of the nuclear medicine QC workflow included multiple steps. First, secondary destinations were setup to send images from each imaging console. Second, appropriate DICOM tags were selected to facilitate image assignment from each imaging unit to the appropriate device, worksheet, and phantom within the software. Standardized QC protocols were created for bar phantom and ACR phantom QC acquisition per ACR recommendations.^4^ Third and finally, the appropriate worksheet and phantom templates were created and imported to a server accessible to the clinical technologists. Technologists were coached to orient the bar phantom (largest bars in the top right) and ACR phantom (smallest sphere at 12 o’clock position) to provide consistent images. To log routine QC data, technologists accessed an interface to the software from a workstation, found the appropriate QC worksheet, and saved the automated results.

### 2.D Image acquisition and reconstruction

Images were acquired using four dual‐head Siemens Symbia gamma cameras with 3/8” crystal thickness (Siemens Healthcare, Erlangen, Germany) and ACR‐recommended quality control procedures. Images using bar phantom 1 were acquired weekly over 18 months for both heads of each gamma camera using the specifications listed in Table 1. Images were acquired extrinsically, per the manufacturer’s specification, and analyzed at matrix sizes of 256 × 256 and 512 × 512 for comparison and to establish the robustness of the technique. To assess the performance of the bar phantom computation over a wider range of bar widths, images of bar phantom 2 and bar phantom 3 were acquired for each head of one camera.

**Table 1:** Bar phantom acquisition parameters.

Isotope	Co‐57
Total Counts	5,000,000
Energy Window	+/‐10% centered on 122 keV
Collimator	Low Energy High Resolution (LEHR)
Matrix Size	256 × 256, 512 × 512
Zoom Factor	1

For the ACR phantom, images were acquired using the same four gamma cameras over the course of 12 months and were all reconstructed on a single Siemens workstation. The phantom was acquired every 3 months for each gamma camera using an ACR‐recommended protocol and as specified in Table 2. Tomographic reconstructions were created with filtered back projection using a Butterworth filter (slope of 6 and cutoff of 0.5) and Chang’s method for attenuation correction (0.15 cm^−1^ coefficient). Reconstructed slices of 3.3‐mm thickness were generated for automated analysis. This is the minimum slice thickness for the matrix size and zoom factor used to acquire the images. Although this slice thickness differs from that described in the ACR protocol (6–9 mm slices), thinner slices were used to demonstrate the robustness of the algorithm. We expect similar results with thicker slices.

**Table 2:** American College of Radiology (ACR) phantom acquisition parameters.

Isotope	Tc‐99m
Injected Activity	10–15 mCi
Energy Window	+/−7.5% centered on 140 keV
Collimator	Low Energy High Resolution (LEHR)
Matrix Size	128 × 128
Zoom Factor	1.45
Acquisition	180° rotation, noncircular orbit, heads in 180^o^ opposed configuration
Number of Views (per head)	64
Total Counts	~32 million
Reconstructed Slice Thickness	3.3 mm
Reconstruction Algorithm	Filtered back projection; with a Butterworth filter (slope = 6, cutoff = 0.5)
Attenuation Correction	Chang‐ 0.15 cm^‐1^ attenuation coefficient

### 2.E Automated quantitative analysis

#### 2.E.1 Bar phantom

Immediately after they were acquired, bar phantom images were sent to the server for analysis. The technologist responsible for QC then accessed the interface from a common workstation, found the appropriate “Bar Phantom” worksheet, and saved the results using the automated analysis. They also manually entered the visually resolvable set of bars on the same worksheet. Thresholds were set within the software that would alert the technologist of a failure if the size of the visually resolvable rods they entered was greater than 3.0 mm, per ACR’s recommended “Satisfactory” criterion for extrinsic resolution testing.^4^

Over the course of the study, the images were also saved to a separate folder to build a research dataset. This dataset was retrospectively reanalyzed using the same analysis templates and exported for better visualization and easier manipulation of the data. A MTF curve was created for each imaging unit using the mean calculated MTF at each bar size across all images acquired on that device. A range and standard deviation were calculated for each bar size for each imaging unit to test the reproducibility of the calculated MTF between different bars sizes.

#### 2.E.2 ACR phantom

For the ACR Phantom, tomographic reconstructions were analyzed. The technologist or physicist then accessed the interface from a common workstation, found the appropriate “ACR Phantom” worksheet, and saved the results using the automated analysis. On the same worksheet, they entered the visually resolvable sphere and section of rods. They also marked whether the uniformity was adequate and if they observed any artifacts within the image.

ACR phantom images were also saved to a separate folder, retrospectively reanalyzed, and the data exported for further analysis. For each acquisition, every slice within the spheres section, rods section, and uniformity section was analyzed using the appropriate template. For each of the sections, a single “best slice” was selected visually and the results shown across varying object size (i.e., CNR by sphere size, modulation by rod size, etc.).

### 2.F Visual evaluation

A subset of the bar phantom images and ACR phantom reconstructions were evaluated visually by three diagnostic medical physicists with varying levels of experience. The original DICOM images were opened in ImageJ and viewed using 300% magnification. The smallest resolvable bar width, sphere, and rod section were reported. The physicists were also asked to mark the single “best slice” to analyze for the spheres and rods section of the ACR phantom. The visual evaluation was then compared to the quantitative methods to estimate appropriate thresholds corresponding to visual resolvability. The physicists had no knowledge of the quantitative results of the images they were reading.

## 3 RESULTS

### 3.A Bar phantom

**Fig. 3:**
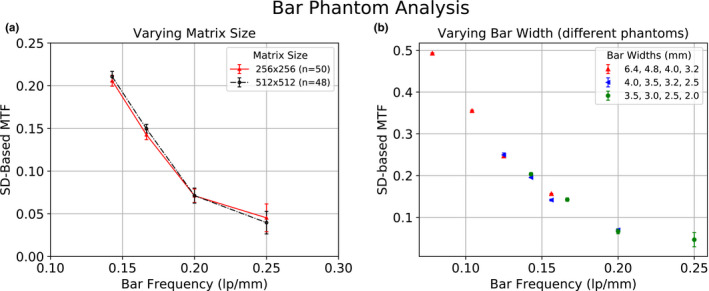
Bar phantom analysis from a representative sample of acquisitions. (a) Results from bar phantom 1 showing the modulation transfer function (MTF) from a representative gamma camera for 256 × 256 and 512 × 512 matrix sizes. (b) Results from all three bar phantoms show the MTF across a wider range of bar widths for a single gamma camera. For both plots, error bars indicate the standard deviation across multiple acquisitions.

**Fig. 4:**
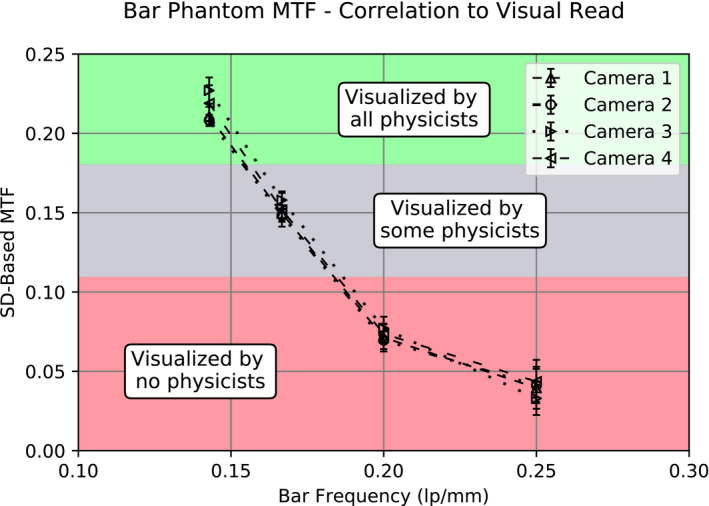
Correlation of the quantitative bar phantom analysis results to a visual read by three medical physicists. All physicists agreed that the 3.5 mm bars were visible and that the 2.5‐ and 2.0 mm bars were not visible. However, there was mixed agreement regarding the visibility of the 3.0 mm bars.

Representative results from the bar phantom MTF analysis are seen in Fig. 3. The SD‐based MTF algorithm was shown to be consistent, irrespective of matrix size [Fig. 3(a)]. For all cameras, there was complete separation in MTF values between the 3.5 and 3.0 mm, as well as the 3.0 and 2.5 mm bars. However, overlap was observed between the 2.5 and 2.0 mm bars. These results are expected given that the MTFs for these bar sizes are below the lower limit of validity of the Hander method (0.1). The sensitivity of the algorithm as a function of frequency response, for a single camera, was determined using the three previously described bar phantoms. Areas where the phantom bar widths overlapped (bar sizes 3.5, 3.2, 3.0, 2.5 mm) were found to have good agreement in the calculated MTF [Fig. 3(b)]. To demonstrate the feasibility of using this algorithm routinely for clinical quality control, images from individual cameras were analyzed over the study period to determine statistical limits on the MTF as a function of bar size. For the subset of images that were evaluated visually, each physicist reported the same bar size as visible across all images (i.e., physicist A reported 3.5 mm bars for all images). However, across physicists, different values were reported (i.e., physicist A reported 3.5 mm bars while physicist B reported 3.0 mm bars as visible, etc.). The visibility categorizations “visualized by all physicists,” “visualized by some physicists,” and “visualized by no physicists” are shown on an MTF curve created from the subset of bar phantom images (Fig. 4). The “visualized by some physicists” region consisted of the 3.0 mm bars and corresponded to a MTF of approximately 0.11 – 0.18.

### 3.B ACR phantom

#### 3.B.1 Slice selection from a single acquisition

**Fig. 5:**
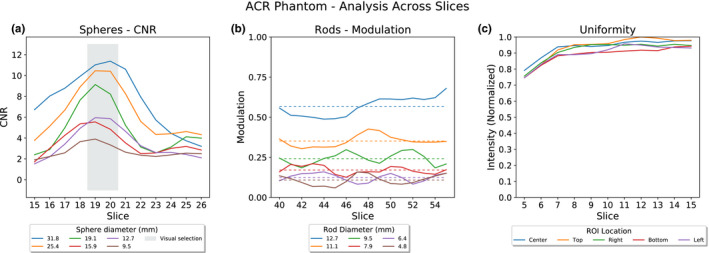
American College of Radiology phantom analysis from a representative sample of acquisitions. (a) Contrast to noise ratio results across slices in the spheres section of the phantom. The shaded region indicates slices that were visually selected as appropriate for analysis. (b) Modulation results from all slices across the rods section of the phantom. Dotted lines show the mean modulation for each rod size as calculated across all slices in the rods section of the phantom and allow for better quantitative separation of the different rod sizes. (c) Uniformity results for all slices in the uniformity section of the phantom.

For the spheres section of each acquisition of the ACR phantom, analysis across axial slices shows a trend of increasing CNR for each sphere until a peak followed by a decline in CNR. This trend in CNR corresponds to the cross‐section of the spheres within each slice; the CNR increases as the spheres appear in the image, peaks around the center of the spheres, and decreases again as the spheres are no longer captured in the slices [Fig. 5(a)]. The light gray box shown in the figure corresponds to slices that were visually selected as appropriate slices to analyze. Figure 5(b) shows that across single slices of the rods section, modulation values can vary depending on slice selection. Averaging the rod modulation across the entire rods section of the phantom, plotted as dotted lines, shows better separation of the mean values for different rod sizes. The analysis across slices within the uniformity section of the phantom generally shows an increase in the mean intensity for all ROIs until a plateau region as shown in Fig. 5(c).

#### 3.B.2 Individual slice analysis and visual comparison

**Fig. 6:**
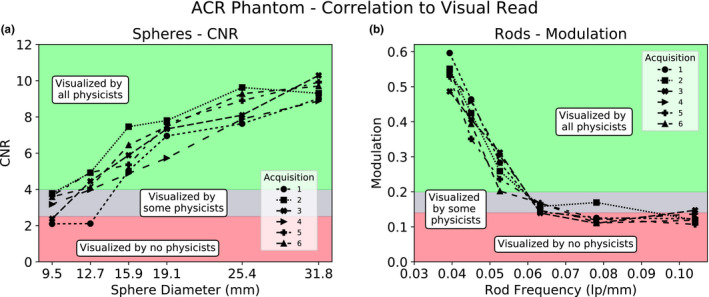
Correlation of a subset of the American College of Radiology Phantom sections to a visual read by three medical physicists. (a) Spheres that were marked as visible by some physicists but marked as not visible by others had contrast to noise ratio values of approximately 2.5–4. (b) Rods that were marked as visible by some physicists but marked as not visible by others had modulation values of approximately 0.15‐0.2.

For every ACR phantom acquisition, the visually selected “best slice” was analyzed for the spheres section. As seen in Fig. 6(a), the values of CNR vary across acquisitions, but also tend to increase with increasing sphere diameter as expected. The correlation between sphere CNR and physicist visibility showed three categorizations as with the bar phantom: spheres “visualized by all physicists,” spheres that are “visualized by some physicists,” and spheres that are “visualized by no physicists.” The “visualized by some physicists” region corresponds to a sphere CNR of roughly 2.5–4. The results from the visual correlation for the spheres section are also shown. The 10‐slice average modulation for each rod size for multiple acquisitions is shown in Fig. 6(b). All acquisitions show decreasing modulation values for increasing rod frequency (decreasing rod size) as expected. The correlation between rod modulation and physicist visibility showed the same categorizations with the “visualized by some physicists” region corresponding to a rod modulation between 0.15 and 0.2. The results from the visual correlation for the rods section are also shown.

## 4 DISCUSSION

### 4.A Bar phantom

#### 4.A.1 Robustness vs matrix size

Changing the image matrix size from 256 × 256 to 512 × 512 was found to have a negligible effect on the results of the SD‐based MTF algorithm for extrinsic planar imaging. This allows for either 256 × 256 or 512 × 512 images to be acquired, depending on the standard protocol recommended by the camera manufacturer, relevant accrediting body, or supervising medical physicist. The matrix size can be changed within a reasonable range, without fear of disrupting the results of the algorithm.

#### 4.A.2 Robustness to bar spacing

Results from the frequency response of a single imaging unit show that the algorithm works well across different bar widths, allowing for the use of any bar phantom that allows for the sampling of at least seven line pair periods.^13^ This flexibility allows clinical sites to use whichever bar phantom they have available for their gamma cameras, with an ideal phantom having at least one bar width that is below the camera’s resolution as reported by the manufacturer.

#### 4.A.3 Quantitative analysis and visual correlation

The smallest resolvable bar width reported by physicists was different, reinforcing that the results of a visual test can vary, even across experienced medical physicists. The MTF curves generated from the SD‐based calculation show continuity across a wide range of clinically relevant spatial frequencies and good separation of MTF values for varying bars, within the statistical differences found for a given bar size. This makes it relatively straightforward to set quantitative thresholds on the MTF values for each bar. For example, for the 512 × 512 matrix size, the 3.5‐mm‐bar MTF should be within μ ± 3σ limits (0.22 ± 0.03) in most cases. These thresholds can be set for each bar to monitor the MTF across multiple frequencies. Alternatively, a linear interpolation of MTF values across the bar widths can be used to estimate the bar width for an MTF that correlates to a smallest “visible bar width.” For instance, since the “visualized by some physicists” region for the bar phantom test was found to be somewhere between 0.11 and 0.18, a threshold around MTF = 0.12 could be set, requiring the interpolated bar width at that MTF to be less than 3.0 mm. These quantitative measures provide a more objective test of the system’s planar spatial resolution. Furthermore, they provide higher sensitivity to subtle changes in camera performance since they are not restricted to the resolution of the four bar frequencies found in the bar phantom and cannot be confounded by reader differences.

### 4.B ACR phantom

#### 4.B.1 Slice selection

The spheres section results indicate that slice selection, along with phantom positioning with respect to the axis of rotation, can influence the quantitative results. However, it should be noted that the visually selected slices for analysis were in all cases near the CNR peak that corresponds to the center of the spheres. We expect that the user performing the test would select a visually appropriate slice to analyze, and the results would reflect what is resolvable within that slice.

For the rods section, slice selection is less straightforward. The calculated modulation shows significant variability based on which individual slice is analyzed. This is likely because each rod has slight fluctuations in its alignment with the reconstructed image matrix because of the coarse pixel sampling inherent to SPECT reconstruction. Averaging the results of the modulation across ten slices shows much more consistent results across multiple acquisitions and has the benefit of analyzing a larger section of the phantom containing the rods. While averaging across multiple slices represents an axial slice that has reduced noise compared to any individual slice, summing of 10‐12 slices has been suggested by both the ACR^4^ and the AAPM^10^ for visual assessment in initial acceptance, routine technologist, and annual physics testing.

For the uniformity test, slice selection affects the calculated percent deviation for all the ROIs, with no discernible trend. Averaging the results across multiple slices can produce an aggregate uniformity measurement for the phantom, but this metric may not be particularly useful for slight nonuniformities.

#### 4.B.2 Phantom rotation

While the effects of ACR phantom rotation were not explicitly studied, the phantom templates expect the phantom to be in a certain orientation. As such, we expect the ACR phantom analysis to be sensitive to rotations of the phantom. With that in mind, the QA technologists have been able to reproducibly place the phantom over the course of this study (26 acquisitions).

#### 4.B.3 Quantitative analysis and visual correlation

As with the bar phantom, the smallest resolvable sphere and rods sections reported by physicists varied. For the spheres section, a CNR of 2.5–4 corresponded well to the “visualized by some physicists” set of spheres. CNR or SNR values of 4–5 have been reported in the past as correlating well to visually resolvable, indicating that this measurement of CNR is well supported historically.^19^, ^21^ For the rods, modulation of around 0.15‐0.2 was the “visible by some physicists” region, which is a similar range as reported for the bar phantom MTF. As with the bar phantom template, quantitative limits can be set on each individual sphere or rod section to more sensitively track tomographic CNR and resolution.

Uniformity analysis can be used to identify phantom images with no‐ or poor attenuation correction. Visual analysis does not indicate that our single‐slice analysis is consistent or sensitive enough to be reliable test of uniformity. Conceivably, it could be used to mark certain slices for a closer visual inspection. Furthermore, it is possible that averaging percent deviation results across the entire uniformity section of the phantom can provide a relevant measure of aggregate uniformity.

### 4.C Limitations

As described earlier, the ROIs for both the ACR and bar phantom are placed using geometric considerations of the phantom, and phantom images are expected to have a certain orientation. The template is built to handle translations of the phantom but is likely highly sensitive to rotations. This was done to limit the computational load and increase the efficiency of testing. For both phantoms, however, this forces the phantom to be placed on the scanner in a consistent manner. Furthermore, for the bar phantom, each quadrant of the detector face will only be tested by the same quadrant of the phantom, and the template design also limits x‐ and y‐directional testing of the system’s spatial resolution.

Additionally, the uniformity test employed by our template is not optimized for the types of artifacts (i.e., ring or bullseyes) commonly found in SPECT imaging. Other methods for uniformity that have been suggested previously target these specific artifacts,^14^, ^16^ but use methods that are either complex, computationally expensive, or not yet developed in QC‐Track. We envision this test for uniformity to provide some quantitative metric that may be useful for assessing the correctness of attenuation correction but believe that a visual assessment is still necessary for evaluation of subtle artifacts.

### 4.D Routine use and clinical feedback

Our method has been user‐friendly enough to have been adopted by technologists with minimal additional training. It has been proven to be robust and efficient in a routine clinical setting. Technologists that regularly use the automated analysis module report that they appreciate the module as it simplifies analysis and saves time.

### 4.E Future work

If ROI misalignment due to phantom rotation is found to be a significant usability issue, we plan to incorporate a coregistration algorithm that aligns the ROIs to each phantom acquisition. Additional functionality is desired to recommend the “best slice” for analysis of the spheres section and to analyze the interior rods of the ACR phantom rods section. We are currently developing templates to automate the analysis of uniformity floods using the NEMA algorithm and to calculate center of rotation misalignment. Additionally, we hope to design a template that allows the bar phantom orientation on the detector to be changed weekly, to better characterize the extrinsic resolution of the entire detector.

## 5 CONCLUSION

Here we have presented a method for automated quantitative quality control for gamma cameras that maintains the efficiency of visual analysis while increasing the sensitivity and consistency of the test. SD‐based MTF analysis of the four‐quadrant bar phantom shows good statistical separation of the MTF values between bar widths, allowing quantitative thresholds to be set for each imaging unit. Likewise, CNR analysis of the ACR phantom spheres section and modulation analysis of the rods section provides good quantitative separation of the results between different sized objects, and correlates well with visual analysis, allowing for a more reliable evaluation of SPECT performance. While the methods described in this paper are like other methods proposed previously, our workflow is structured in a way that allows for easy clinical implementation and routine use for both technologists and physicists. Practically speaking, our methodology can be used to improve routine clinical nuclear medicine QC programs as quantitative metrics provide more consistent, sensitive measurements than visual analysis.

## CONFLICT OF INTEREST

Tutku E. Tazegul, Carl Snyder, and Angela Snyder are employees of Atirix Medical Systems Inc. Atirix sells the QC‐Track system for imaging QC and provided funding for this research.

## AUTHOR CONTRIBUTIONS

This project was a collaborative effort. T.T, A.P, A.S, C.S, and P.C. all contributed to conceptual formulation of the idea. T.T., A.S, and C.S carried out the initial algorithm design and implementation, and data analysis. A.P and P.C. acquired data, coordinated clinical implementation, and interpreted results. T.T. took the lead in writing the manuscript. All authors contributed significantly to manuscript revisions, both before and after peer review. All authors reviewed and approved the final submitted version of the manuscript.

## References

[1] American College of Radiology . *Quality Control: Nuclear Medicine (Revised 12‐12‐19).* https://accreditationsupport.acr.org/support/solutions/articles/11000061046‐quality‐control‐nuclear‐medicine‐revised‐12‐12‐19‐. Accessed February 27, 2020

[2] Intersocietal Accreditation Commission . *The IAC Standards and Guidelines for Nuclear/PET Accreditation* https://www.intersocietal.org/nuclear/standards/IACNuclearPETStandards2016.pdf. Accessed March 2, 2020.

[3] Murphy PH . Acceptance testing and quality control of gamma cameras, including SPECT. J Nucl Med Off Publ Soc Nucl Med. 1987;28:1221–1227. 3496436

[4] American College of Radiology . *Phantom Testing: Nuclear Medicine (Revised 12‐12‐19).* https://accreditationsupport.acr.org/support/solutions/articles/11000062798‐phantom‐testing‐nuclear‐medicine‐revised‐12‐12‐19‐. Accessed February 27, 2020

[5] Nichols KJ , DiFilippo FP , Palestro CJ . Texture analysis for automated evaluation of Jaszczak phantom SPECT system tests. Med Phys. 2019;46:262–272. 30418674 10.1002/mp.13289

[6] American Association of Physicists in Medicine . *AAPM Report 9:* *Computer‐Aided Scintillation Camera Acceptance Testing* https://www.aapm.org/pubs/reports/RPT_09.pdf. Accessed March 2, 2020.

[7] Nelson JS , Christianson OI , Harkness BA , et al. Improved nuclear medicine uniformity assessment with noise texture analysis. J Nucl Med Off Publ Soc Nucl Med. 2014;55:169–174. 10.2967/jnumed.113.125450 24212975

[8] American Association of Physicists in Medicine . *AAPM Report 22: Rotating Scintillation Camera SPECT Acceptance Testing and Quality Control.* https://aapm.org/pubs/reports/RPT_22.pdf. Accessed March 5, 2020.

[9] Graham LS , Fahey FH , Madsen MT , van Aswegen A , Yester MV . Quantitation of SPECT performance: report of task group 4, nuclear medicine committee. Med Phys. 1995;22:401–409. 7609720 10.1118/1.597605

[10] American Association of Physicists in Medicine . *AAPM Report 177: Acceptance Testing and Annual Physics Survey Recommendations for Gamma Camera, SPECT, and SPECT/CT Systems.* https://www.aapm.org/pubs/reports/RPT_177.pdf. Accessed March 5, 2020.

[11] Hasegawa BH , Kirch DL , LeFree MT , Vogel RA , Steele PP , Hendee WR . Quality control of scintillation cameras using a minicomputer. J Nucl Med Off Publ Soc Nucl Med. 1981;22:1075–1080. 7310516

[12] Hander TA , Lancaster JL , Kopp DT , Lasher JC , Blumhardt R , Fox PT . Rapid objective measurement of gamma camera resolution using statistical moments. Med Phys. 1997;24:327–334. 9048375 10.1118/1.597928

[13] Hander TA , Lancaster JL , McDavid W , Kopp DT . An improved method for rapid objective measurement of gamma camera resolution. Med Phys. 2000;27:2688–2692. 11190951 10.1118/1.1328083

[14] Madsen MT . A method for quantifying SPECT uniformity. Med Phys. 1997;24:1696–1700. 9394276 10.1118/1.597956

[15] Vickery A , Jørgensen T , Nijs R . NEMA NU‐1 2007 based and independent quality control software for gamma cameras and SPECT. J Phys Conf Ser. 2011;317:012023.

[16] Hirtl A , Bergmann H , Knausl B , Beyer T , Figl M , Hummel J . Technical Note: Fully‐automated analysis of Jaszczak phantom measurements as part of routine SPECT quality control. Med Phys. 2017;44:1638–1645. 28186647 10.1002/mp.12150

[17] DiFilippo FP . Technical Note: Automated quantitative analysis of planar scintigraphic resolution with the ACR SPECT phantom. Med Phys. 2018;45:1118–1122. 29385653 10.1002/mp.12779

[18] DiFilippo FP . Assessment of PET and SPECT phantom image quality through automated binary classification of cold rod arrays. Med Phys. 2019;46:3451–3461. 31115055 10.1002/mp.13616

[19] Cherry S , Sorenson J , Phelps M . CH. 15: Image quality in nuclear medicine In: Physics in Nuclear Medicine, 4th edn Saunders; 2012.

[20] American College of Radiology . *Phantom Testing: CT (Revised 12‐12‐19).* https://accreditationsupport.acr.org/support/solutions/articles/11000056197‐phantom‐testing‐ct‐revised‐12‐12‐19‐. Accessed March 7, 2020.

[21] Rose A . The sensitivity performance of the human eye on an absolute scale*. J Opt Soc Am. 1948;38:196–208. 18901781 10.1364/josa.38.000196

